# Optimization of extraction condition for platycodin D from *Platycodon grandiflorum* root and verification of its biological activity

**DOI:** 10.1002/fsn3.3585

**Published:** 2023-07-30

**Authors:** Jihyeon Jung, Yeon Jin Cho, Minju Jeong, Seung‐Su Lee, Jong Hun Kim, Jong‐Eun Kim, Nara Kim, Jiyun Lee, Jung Han Yoon Park, Ki Won Lee, Sung‐Young Lee

**Affiliations:** ^1^ Department of Agricultural Biotechnology Seoul National University Seoul South Korea; ^2^ Bio‐MAX Institute, Seoul National University Seoul South Korea; ^3^ BOBSNU Co., Ltd. Seoul Techno Holdings, Inc. Subsidiary Company Suwon South Korea; ^4^ Department of Food Science and Biotechnology Sungshin Women's University Seoul South Korea; ^5^ Department of Food Science & Technology Korea National University of Transportation Jeungpyeong Republic of Korea; ^6^ Advanced Institute of Convergence Technology Seoul National University Seoul South Korea; ^7^ Research Institute of Agriculture and Life sciences Seoul National University

**Keywords:** platycodin D, *Platycodon grandiflorum*, response surface methodology

## Abstract

Platycosides, major components of *Platycodon grandiflorum* (PG) extract, have been implicated in a wide range of biological effects. In particular, platycodin D (PD) is a well‐known main bioactive compound of Platycosides. Despite the biological significance of PD, optimization of extract condition for PD from PG root has not been well investigated. Here, we established the optimum extraction condition as ethanol concentration of 0%, temperature of 50°C, and extraction time of 11 h to obtain PD‐rich *P. grandiflorum* extract (PGE) by using response surface methodology (RSM) with Box–Behnken design (BBD). The 5.63 mg/g of PD was extracted from the PG root in optimum condition, and this result was close to the predicted PD content. To analyze the biological activity of PGE related to mucin production, we demonstrated the inhibitory effect of PGE on PMA‐induced hyperexpression of MUC5AC as well as ERK activation, a signal mediator of MUC5AC expression. Moreover, we showed that PGE had expectorant activity in mice. These results indicated that PGE had sufficient functions as a potential mucoregulator and expectorant for treating diverse airway diseases. Additionally, we confirmed that PGE had antioxidant activity and inhibited LPS‐induced proinflammatory cytokines, TNF‐α, and IL‐6. Taken together, PGE derived from novel optimizing conditions showed various biological effects, suggesting that PGE could be directly applied to the food industry as food material having therapeutic and preventive potential for human airway diseases.

## Graphical abstract

Establishment of optimum condition for PD‐rich *Platycodon grandiflorum* extract (PGE). Analyzed biological activity of PD‐rich *Platycodon grandiflorum* extract (PGE).

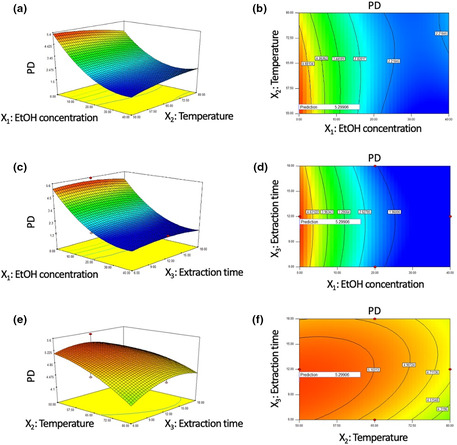

## 1 INTRODUCTION

*Platycodon grandiflorum* (PG) is a perennial plant widely used as a traditional oriental medicine. Especially the root of PG has been known to have biological effects on pulmonary diseases (Ryu et al., 2014), obesity (Chung et al., 2012), inflammation (Jang et al., 2013), cancer (Yim et al., 2016), oxidative stress (Jang et al., 2011), and hepatic steatosis (Noh et al., 2010). Some studies have revealed that the main components that elicit the biological activities in the root of PG are the Platycosides, the triterpenoid saponin family in PG, including platycodin D, platycodin D3, platycoside E, and polygalacin D (Noh et al., 2010). These saponins can be applied as additives in the food and cosmetic industries, as wetting agents in the agriculture industry, and as adjuvants in the pharmaceutical industry (San Martin & Briones, 1999).

In particular, platycodin D (PD), a significant platycoside in PG, has also been reported to have expectorant effects against mucus excretion (Shin et al., 2002). Pulmonary mucin is important in defense against inhaled irritants such as dust, bacteria, and allergens. In particular, hypersecretion or hyperproduction of MUC5AC, a significant component of airway mucus, reflects hypersecretion or hyperproduction of pulmonary mucus, commonly observed in diverse pulmonary diseases.

Despite the biological significance of PD, the studies on the extraction method to obtain high PD content from PG roots have not been well investigated. In this study, we demonstrated the optimum extraction method to obtain PD‐rich *P. grandiflorum* extract (PGE) with the solvent available in the food industry using response surface methodology (RSM). RSM is a statistical tool that drives optimum conditions based on the number of experimental trials formed from several independent variables. There are various experimental designs, such as central composite design (CCD), Taguchi design, Placket–Burman design, and Box–Behnken design (BBD), to determine experimental parameters for economical, fast, and effective optimization (Taşpınar et al., 2021). BBD can minimize experimental trials compared to other designs and performs well in optimizing input variables (Sultana et al., 2020).

In this study, we evaluated the optimum extraction conditions using RSM modeling to maximize the yield of PD from the PG root. Additionally, we verified the biological effects of the PD‐rich *P. grandiflorum* extract (PGE) as a potential mucoregulator and expectorant.

## 2 MATERIALS AND METHODS

### 2.1 Chemicals and reagents

Ethanol (purity 95%) used for extraction was purchased from Daehan Ethanol Life Co., Ltd. PD and platycodin D3 standards for HPLC analysis were purchased from Wuhan ChemFaces Biochemical Co., Ltd.

### 2.2 Preparation of *P. Grandiflorum*

Hot‐air‐dried *P. Grandiflorum* (PG) root was purchased from the market (Sejong‐nongsan). *P. grandiflorum* was finely ground using a pulverizer (Mill powder tech®) and sieved through a 500 μm stainless steel sieving mesh. The PG powder was stored in a dry chamber at room temperature.

### 2.3 Experimental design

Box–Behnken design (BBD) is a spherical second‐order design based on three levels of independent variables. BBD can be conducted to verify the interactions between the variables with a central point and middle points of the cube's edges restricted on the sphere (Kumar et al., 2008). This study used Design Expert V7.0 statistical software (StatEase Inc.) to determine optimum conditions and investigate the effect of three variables. A total of 15 experimental runs, including triplicate of the central point, are shown in Table 1 for the three‐level and three‐factorial BBD.

**TABLE 1:** Box–Behnken design with predicted and actual values.

Trials	*X*_1_: Ethanol concentration (%)	*X*_2_: Temperature (°C)	*X*_3_: Extraction time (h)	Actual value (mg/g)	Predicted value (mg/g)
1	20 (0)	50 (−1)	6 (−1)	2.06	2.18
2	0 (−1)	50 (−1)	12 (0)	5.53	5.30
3	40 (1)	50 (−1)	12 (0)	1.72	1.63
4	20 (0)	50 (−1)	18 (1)	1.80	2.02
5	0 (−1)	65 (0)	6 (−1)	4.68	4.80
6	40 (1)	65 (0)	6 (−1)	1.83	1.80
7	20 (0)	65 (0)	12 (0)	2.35	2.55
8	20 (0)	65 (0)	12 (0)	2.74	2.55
9	20 (0)	65 (0)	12 (0)	2.58	2.55
10	0 (−1)	65 (0)	18 (1)	4.84	4.87
11	40 (1)	65 (0)	18 (1)	2.17	2.05
12	20 (0)	80 (1)	6 (−1)	2.13	1.91
13	0 (−1)	80 (1)	12 (0)	4.48	4.58
14	40 (1)	80 (1)	12 (0)	2.17	2.42
15	20 (0)	80 (1)	18 (1)	2.50	2.37

The experimental data were fitted to the following second‐order polynomial model equation:

$$Y=\beta_{0}+\sum_{i=1}^{3}\beta_{i}X_{i}+\sum \sum_{i<j}^{3}\beta_{\mathrm{ij}}X_{i}X_{j}+\sum_{i=1}^{3}\beta_{\mathrm{ii}}X_{i}^{2}$$

Where *Y* is the predicted response, *X*_*i*_ and *X*_*j*_ are the independent variables, and *β*_0_, *β*_*i*_, *β*_*ii*_, and *β*_*ij*_ are the regression coefficients for intercept, linear, quadratic, and interaction terms, respectively.

### 2.4 Sample preparation and LC–MS/MS analysis

PD from *P. grandiflorum* extract was analyzed on an LC–MS system (Agilent Technologies 6410 Triple quadrupole) equipped with a heated ESI interface. The sample solution was prepared by dissolving *P. grandiflorum* extract in MeOH/DW (1:3, v/v) solution at 1000 ppm. A C_18_ reversed‐phase column (Kromasil, 3.0 μm, 3.0 mm × 150 mm; Merck) was used for separation. The column temperature was 26°C. The mobile phase was a binary gradient with 0.1% formic acid in water (A) and 0.1% formic acid in acetonitrile (B). The gradient elution was 0 min, 10% B; 1–6 min, 15% B; 7–20 min, 15% B; 21–30 min, 25% B; 31–35 min, 100% B; and the flow rate was 0.4 mL/min. The MS detector was operated in negative ion mode with the following settings: capillary voltage of 4 kV, gas temperature of 350°C, nebulizer of 40 psi, and fragmentor of 135 V.

### 2.5 Biological activity

#### 2.5.1 Antioxidant activity

For the measurement of antioxidant activity, 2–2′‐azinobis‐(3‐ethylbenzothiazoline‐6‐sulfonic acid) (ABTS) assay and hydroxyl radical‐mediated oxidation of protein as the cytoprotective effect was performed with slight modification (Cho et al., 2020; Lee, Jang, et al., 2020). ABTS radical solution was prepared by mixing 20 mL of 7.4 mM ABTS and 2.45 mM potassium persulfate. After the ABTS radical solution stood in a dark environment at room temperature for 16 h, the ABTS solution was diluted with distilled water to obtain an absorbance of 0.7 ± 0.02 units at 734 nm using the spectrophotometer. A 180 μL of ABTS radical solution was added to 20 μL of PGE in 96 plates and stored for 10 min in a dark condition. Then, the absorbance was taken at 734 nm.

The protective effect of PGE on hydroxyl radical‐mediated oxidation was measured based on Galano et al. (2010). A solution of ascorbic acid (0.8 mM)/EDTA (0.4 mM)/ferrous ammonium sulfate (0.4 mM) was prepared in phosphate buffer, pH 7.4, and bovine serum albumin (BSA) was dissolved in it. A 235 μL of the solution was added to 15 μL of PGE or water, and 250 μL of 1% BSA was mixed. Hydroxyl radical was generated by adding 15 μL of 2% H_2_O_2,_ and it was replaced by water for control. After 3 h of incubation at room temperature, 250 μL of 20% trichloroacetic acid was added, and the mixture was centrifuged at 2000 *g* for 30 min at 4°C. The supernatant was discarded, and the pellet was resuspended with 500 μL of 0.1 M NaOH. It was mixed 1:1 with loading buffer and heated at 100°C for 1 min. The protein samples were loaded in a 12% polyacrylamide gel and electrophoresed at 100 V. After running, gels were stained with 0.2% Coomassie brilliant blue R for 1 h and destained with deionized water.

#### 2.5.2 Cell culture

NCI‐H292 cells, a human lung mucoepidermoid carcinoma cell line, were purchased from the Korean Cell Line Bank (KCLB). The cell medium contained RPMI 1640 (Welgene), 10% fetal bovine serum (FBS) (Gibco), and 1% (w/v) antibiotic penicillin/streptomycin (Hyclone) for cell growth. Cells were cultured in a T75 cell culture flask in a CO_2_ incubator at 5% CO_2_/air‐humidified atmosphere at 37°C. For serum deprivation, confluent cells (5 × 10^5^ cells/well in a six‐well plate) were washed twice with PBS and recultured in RPMI 1640 with 0.2% fetal bovine serum for 24 h. RAW264.7 murine macrophage cells were purchased from the Korean Cell Line Bank. Cells were cultured in Dulbecco's modified Eagle medium (Welgene) containing 10% fetal bovine serum (FBS) (Gibco) and 1% penicillin/streptomycin (Corning). RAW264.7 murine macrophage cells were maintained in a 5% CO_2_ humidified chamber at 37°C.

#### 2.5.3 Real‐time quantitative PCR analysis

Total RNA was isolated using a Total RNA Extraction kit (MGmed), and 1.5 μg of total RNA was reverse transcribed to cDNA in a 20 μL reaction system using a cDNA Synthesis kit (Takara Bio) according to the manufacturer's protocols. qRT‐PCR was performed using the IQ Sybr Green SuperMix (Biorad) and used the following qRT‐PCR conditions: pre‐denaturation at 95°C for 10 min, followed by 44 cycles of amplification of 95°C for 10 s, and 65°C for 30 s. The oligonucleotide primer sequences were as follows: MUC5AC (forward: 5′‐TGT TCT ATG AGG GCT GCG TCT‐3′ and reverse: 5′‐ATG TCG TGG GAC GCA CAG A‐3′) and β‐actin (forward: 5′‐TCC TCA CCC TGA AGT ACC CCA T‐3′ and reverse: 5′‐ AGC CAC ACG CAG CTC ATT GTA‐3′). Relative quantities of MUC5AC mRNA were obtained using a comparative cycle threshold method and normalized using β‐actin as an internal control. Fold change in gene expression was calculated by using the 2^−ΔΔ*C*t^ method.

#### 2.5.4 Cell viability

To examine the cytotoxicity of PGE on NCI‐H292 and RAW264.7 cells, cell viability was measured by MTT assay and Cell Titer Glo assay, respectively. NCI‐H292 cells were seeded (2 × 10^4^ cells per well) in a 96‐well plate, incubated for 48 h, and then treated with various concentrations of PGE (0–400 μg/mL). After incubation for 24 h, 10 μL of MTT (3‐(4, 5‐dimethylthiazol‐2‐yl)‐2, 5‐diphenyltetrazolibromide) solution (Affymetrix USB products) was added, and cells were incubated for 3 h at 37°C in a 5% CO_2_ incubator. Absorbance was measured at 570 nm. RAW264.7 cells were seeded (4 × 10^4^ cells per well) in a 96‐well white luminescence plate and incubated for 24 h. Cells were then starved in serum‐free DMEM for 24 h, followed by incubation with various concentrations of PGE (0–100 μg/mL). After a 24 h incubation, a luminescent reagent was added to each well. The plate was shaken on an orbital shaker for 1 min and incubated at RT for 10 min. Luminescence was measured using a Varioskan Lux Multimode microplate reader (Thermo Fisher Scientific).

#### 2.5.5 Dot blot assay

Dot blot assay was performed using whole‐cell extracts prepared by disrupting cells in RIPA buffer, including protease inhibitor cocktail (Thermo Scientific). The protein concentration was measured using bicinchoninic acid assay (Peirce Biotechnology) as described in the manufacturer's manual. Protein lysate (5–10 μg) was loaded onto a nitrocellulose membrane, blocked with 5% of skim milk, and hybridized with anti‐MUC5AC antibody (clone 45 M1; Invitrogen) overnight at 4°C. A horseradish peroxidase‐conjugated secondary antibody (Peirce Biotechnology) was used, and the signal was detected with a chemiluminescence reagent (Biorad).

#### 2.5.6 Western blot analysis

Harvested cells were disrupted with RIPA buffer, including protease inhibitor cocktail (Thermo Scientific). The protein concentration was measured using a bicinchoninic acid assay (Peirce Biotechnology) as described in the manufacturer's manual. Protein lysate (10–20 μg) was subjected to SDS–polyacrylamide gel electrophoresis and electrophoretically transferred to a polyvinylidene difluoride membrane (Millipore). After blotting, the membrane was incubated overnight with a specific primary antibody at 4°C. Protein bands were visualized by a chemiluminescence detection kit (Biorad) after hybridization with a horseradish peroxidase‐conjugated secondary antibody (Peirce Biotechnology). For Western blotting, anti‐phospho‐ERKs (Cell signaling), anti‐ERKs (Cell signaling), and monoclonal anti‐β‐actin (Sigma) were used.

#### 2.5.7 Enzyme‐linked immunosorbent assay (ELISA)

RAW264.7 murine macrophage cells were cultured in 12‐well plates for 24 h and then starved in serum‐free DMEM for 24 h. The cells were pretreated with PGE (100 μg/mL) for 1 h and then treated with LPS (10 ng/mL) for 24 h. Media were centrifuged, and supernatants were collected and stored at −80°C. Levels of TNF‐α and IL‐6 in cell culture supernatant were analyzed using mouse TNF‐α kits and mouse IL‐6 ELISA kits according to the manufacturer's protocol (R&D Systems). The absorbance at 450–570 nm was measured using the Varioskan Lux Multimode microplate reader (Thermo Fisher Scientific).

### 2.6 The expectorant activity

#### 2.6.1 Animals

Male BALB/c mice (8‐week‐old) were purchased from Koatech Company, Korea. They were acclimatized to the laboratory for 7 days in a light‐ and temperature‐controlled room with a 12 h dark–light cycle and fed with commercial pelleted feed from Teklad Global Diets. The animal study was performed according to the international rules considering animal experiments and the internationally accepted ethical principles for laboratory animal use and care.

#### 2.6.2 The tracheobronchial secretion assay in mice

After 7 days of adaption, mice were randomly divided into four groups (*n* = 5). Group 1 was administered with distilled water (10 mL/kg/day, p.o.) as a vehicle of PGE and Ambroxol, Group 2 animals were administered with Ambroxol at 250 mg/kg, Group 3–4 animals were treated with 100 and 400 mg/kg of PGE for 7 days. After 30 min of the last administration, mice were injected with 5% phenol red solution (500 mg/kg) intraperitoneally. Another 30 min later, mice were dissected to remove the trachea (P212013). The trachea was placed into 1 mL of saline, ultrasonicated for 30 min, and centrifuged at 10,000 rpm for 5 min. 1 N NaOH was added, and the optical density of the mixture was measured at 558 nm using a SpectraMax M2 microplate reader (Molecular Devices).

### 2.7 Statistical analysis

The analysis of variance (ANOVA) indicated the significance and fitness of the model and independent variables. *F* and *p* values reflect the significance, and the determination regression (*R* ^2^), adjusted *R* ^2^, and similarity coefficient (CV) contribute to evaluating the suitability of the model (Row & Park, 2013). ANOVA was performed to determine the significance with descriptive statistical analysis such as *p*‐value, *F*‐value, sum of squares (SS), coefficient estimate (CE), standard error (SE), and determination coefficient (*R* ^2^) evaluated by experimental data. Data are expressed as mean values ± standard deviation (SD). For multiple comparisons, analysis of variance was used, followed by Tukey. Statistical analysis was performed with SPSS (version 26). Differences were regarded as significant if the value of *p* < .01.

## 3 RESULTS & DISCUSSION

### 3.1 Optimization using response surface methodology

Response surface methodology (RSM) is one of the effective statistical methods for optimization considering independent factors and interactions between them. RSM is an effective tool used in various industries, including food, to devise efficient methods (Liyana‐Pathirana & Shahidi, 2005). It validates a multivariate equation utilizing a minimum of experiments and quantitative data (Quanhong & Caili, 2005). Especially, one of the RMS designs, the Box–Behnken design (BBD) used in the current study, has fewer design points than the central composite design, which means it can be an economical design for the application in industry (Aslan & Cebeci, 2007).

Fifteen experimental trials with three independent variables, including ethanol concentration (*X*_1_), temperature (*X*_2_), and extraction time (*X*_3_), were investigated (Table 1). PD content of PGE ranged from 1.72 to 5.53 mg/g. Based on these results, the correlation between response and independent variables was expressed with the equation as follows;

$$\mathrm{PD}=2.553–1.4548X_{1}+0.0205X_{2}+0.0778X_{3}+0.3741X_{1}X_{2}+0.046X_{1}X_{3}+0.1565X_{2}X_{3}+1.0918X_{1}^{2}–0.1687X_{2}^{2}–0.2653X_{3}^{2}$$

In Table 2, the model obtained by BBD and ethanol concentration variable were statistically significant (*p* < .05). Lack of fit which clarifies the significance of the model, was nonsignificant (*p* > .05). Also, the *R* ^2^ and adjusted *R* ^2^ were .98 and .95, respectively.

**TABLE 2:** Analysis of variance (ANOVA) for the fitted quadratic models.

Source	SS	CE	SE	*F*‐value	*p*‐Value
Model	22.70	2.55	0.16	33.20	.0006
*X*_1_—Ethanol concentration	16.93	−1.45	0.10	222.90	<.0001
*X*_2_—Temperature	0.00	0.02	0.10	0.04	.8419
*X*_3_—Time	0.05	0.08	0.10	0.64	.4611
*X*_1_ *X*_2_	0.56	0.37	0.14	7.37	.0421
*X*_1_ *X*_3_	0.01	0.05	0.14	0.11	.7521
*X*_2_ *X*_3_	0.10	0.16	0.14	1.29	.3076
$X_{1}^{2}$	4.40	1.09	0.14	57.94	.0006
$X_{2}^{2}$	0.11	−0.17	0.14	1.38	.2924
$X_{3}^{2}$	0.26	−0.27	0.14	3.42	.1236
Residual	0.38				
Lack of fit	0.30			2.66	.2846
*R* ^2^	.98				
Adjusted *R* ^2^	.95				

Abbreviations: CE, coefficient estimate; SE, standard error; SS, sum of squares.

**FIGURE 1:**
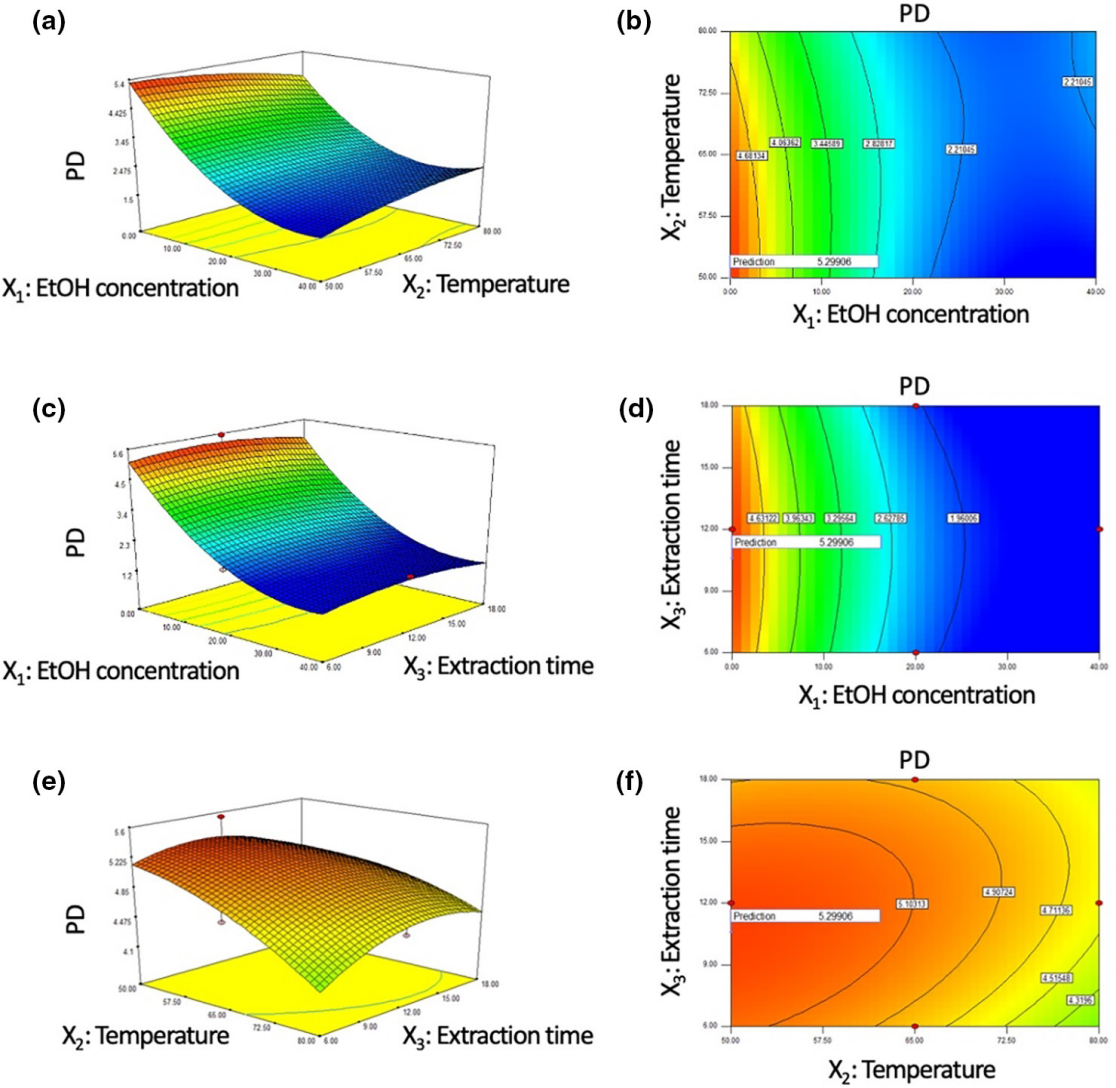
Interaction effect of independent variables on PD.

Three‐dimensional (3D) surface plots and 2D contour plots indicating the relationship between response and independent variables were generated to see graphically how the response changes with the factors. Each graph expressed the correlation of two independent variables after fixing another variable to level 0. The response surface plots for the concentration of extracted PD were constructed according to their fitted model (Figure 1). Figure 1a–d showed the interactive effect between ethanol concentration (*X*_1_) and temperature (*X*_2_), and ethanol concentration (*X*_1_) and time (*X*_3_), respectively. It indicated that ethanol usage is unsuitable for extracting PD from PG. However, the interaction between temperature (*X*_2_) and time (*X*_3_) showed there was a steady increase in PD at definite temperatures (50–80°C), indicating that time has no significant role (Figure 1e,f). Choi et al. (2007) reported that the PD content in *P. grandiflorum* hydrothermal extracts was insignificant with extraction temperature and time longer than 5 h. In the current study, we showed that the *p*‐values of *X*_2_, *X*_3_, and *X*_2_ *X*_3_ exceed .05, indicating that they were not significant, confirmed by the ANOVA system (Table 2). This result showed that extraction times longer than 6 h did not affect PD content, consistent with the previous study (Choi et al., 2007).

Previously, a few extraction methods for PD form PG root, such as ultrasonic extraction (Kwon et al., 2017), mechanochemical‐assisted extraction (Wu & Xi, 2020), fermentation (Lee, Han, et al., 2020), and enzymatic extraction (Li et al., 2012), have been reported. The PD concentration extracted by these methods ranged from 5.4 to 7 mg/g, which is higher than typical solvent extraction. However, it is considered that availability of these methods for direct application to the food industry seems low because of complexity, time, cost, etc. Unlike these methods, the extraction condition optimized in the present study provides a simple way to obtain PD‐rich PGE using solvents that can be directly applied to the food industry.

### 3.2 Verification of model

The optimum conditions obtained from the fitted model were ethanol concentration of 0%, temperature of 50°C, and extraction time of 11 h. The predicted value of PD content was 5.38 mg/g. The optimum condition was performed to verify the fitness, and the experimental value of PD was 5.63 mg/g. The predicted and observed values showed similar results, indicating that the fitted model is suitable for optimizing the extraction method to obtain PD‐rich PGE.

### 3.3 Biological activities of PGE

#### 3.3.1 Antioxidant effects of PGE

**FIGURE 2:**
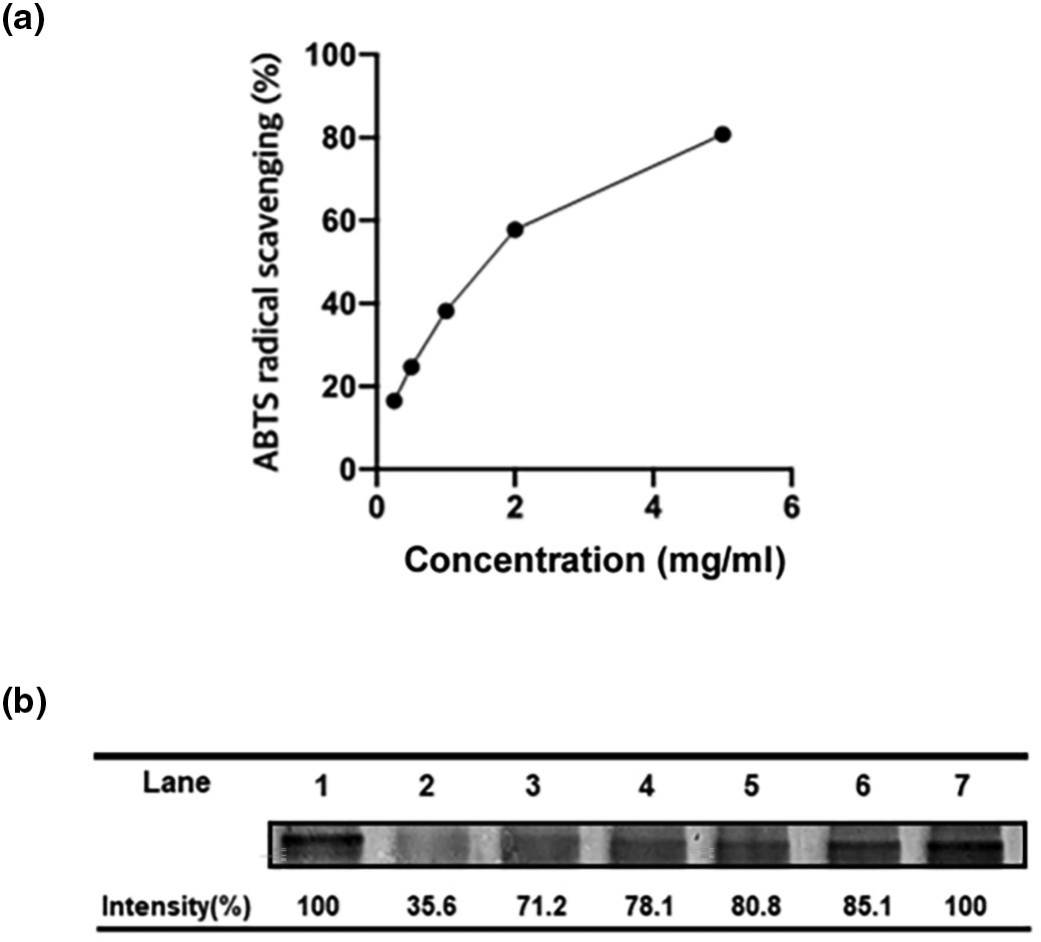
Antioxidant activities of PGE.

Previously, the antioxidant activity of PD was reported (Yuk et al., 2015). To confirm the antioxidant effect of PGE, we performed ABTS free‐radical scavenging assay at various concentrations of PGE as described in Section 2. As shown in Figure 2a, the scavenging activity of PGE toward ABTS increased dose dependently.

Oxidation systems with metal catalyst accelerate the fragmentation of proteins, which can be protected by antioxidants that scavenge H_2_O_2_. Fe^2+^/EDTA‐catalyzed oxidation system expresses the formation of HO·radicals formed by H_2_O_2_, and it causes the degradation of albumin (Kocha et al., 1997). The degradation of albumin by the Fe^2+^/EDTA‐catalyzed oxidation system is indicated in Figure 2b lane 2, and it was recovered by adding PGE in a dose‐dependent manner (Figure 2b lane 3–7). These results suggest that PGE sufficiently protects albumin (BSA) against hydroxyl radical‐mediated protein oxidation.

Taken together, these results suggest that PGE has free radical scavenging ability as an antioxidant.

#### 3.3.2 Cytotoxic effect of PGE

**FIGURE 3:**
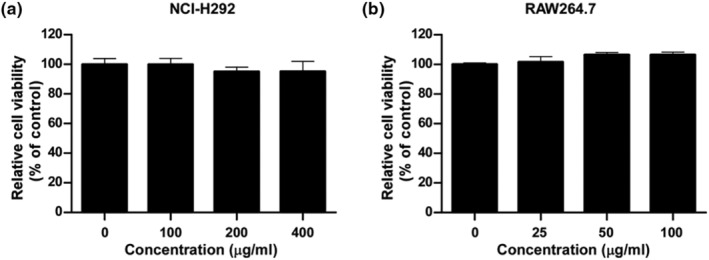
Cytotoxic effect of PGE.

To examine the cytotoxicity of PGE on NCI‐H292 and RAW264.7 cells, 90% confluent NCI‐H292 and RAW264.7 cells were treated with various concentrations of PGE for 24 h and cell viability was assessed by MTT and Cell Titer Glo assay, respectively. The PGE did not show significant cytotoxic effects at concentrations up to 400 μg/mL in NCI‐H292 cells (Figure 3a) and up to 100 μg/mL in RAW264.7 cells (Figure 3b). Therefore, we studied the biological effect of PGE at concentrations of up to 400 μg/mL for NCI‐H292 cells and 100 μg/mL for RAW264.7 cells in cell‐based experiments.

#### 3.3.3 Inhibitory effect of PGE on PMA‐induced MUC5AC mRNA and protein expression

**FIGURE 4:**
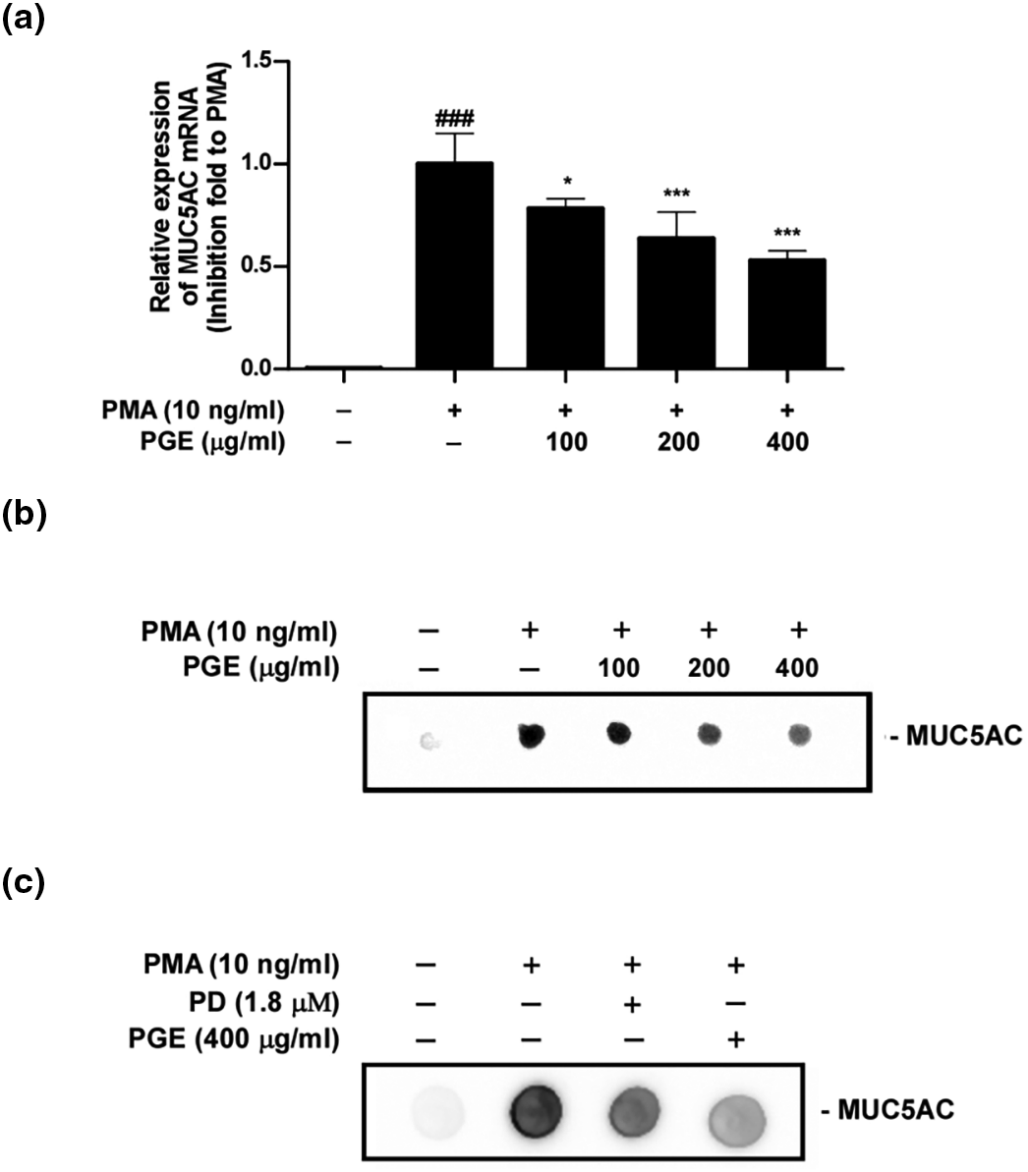
PGE inhibits expression of MUC5AC mRNA and protein.

PMA (phorbol 12‐myristate 13‐acetate) is known to induce the expression of MUC5AC mRNA and protein in NCI‐H292 cells (Hewson et al., 2004). To investigate the inhibitory effect of PGE on PMA‐induced MUC5AC mRNA and protein expression, NCI‐H292 cells were pretreated with various concentrations of PGE for 30 min, followed by incubation with PMA (10 ng/mL) for 24 h. We confirmed that PMA markedly increased the expression of MUC5AC mRNA (Figure 4a) and protein (Figure 4b) compared with the untreated group. PGE significantly inhibited the expression of MUC5AC mRNA (Figure 4a) and protein (Figure 4b) in a dose‐dependent manner.

To verify biological activity on PMA‐induced MUC5AC expression between PGE and PD, we compared the inhibitory effect between 400 μg/mL of PGE and 1.8 μM of PD, which corresponds content of PD for 400 μg/mL of PGE, on PMA‐induced MUC5AC expression. As shown in Figure 4c, 400 μg/mL of PGE more effectively suppressed PMA‐induced MUC5AC expression than 1.8 μM of PD. This result is considered derived from PGE containing bioactive saponins, including PD (Choi et al., 2015; Ryu et al., 2014).

Together, current results are consistent with previous reports that PD inhibited PMA‐induced MUC5AC mucin production in NCI‐H292 cells (Lee et al., 2010).

#### 3.3.4 Inhibitory effect of PGE on PMA‐induced ERK phosphorylation

**FIGURE 5:**
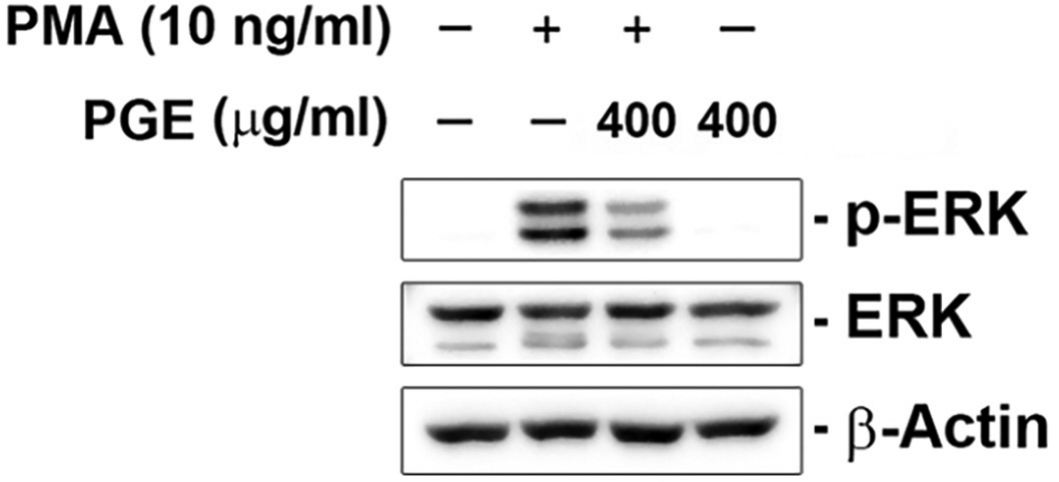
PGE inhibits phosphorylation of ERK.

Previously, it has been reported that PMA activated the phosphorylation of ERK in NCI‐H292 cells. Additionally, ERK pathway has been known as one of the critical signal pathways in mucin production (Hewson et al., 2004). Here, we examined whether PGE inhibits the phosphorylation of ERK induced by PMA. As shown in Figure 5, ERK phosphorylated by PMA was significantly inhibited by PGE. This result suggests that PGE regulates MUC5AC production through the ERK pathway.

#### 3.3.5 Anti‐inflammatory effects of PGE

**FIGURE 6:**
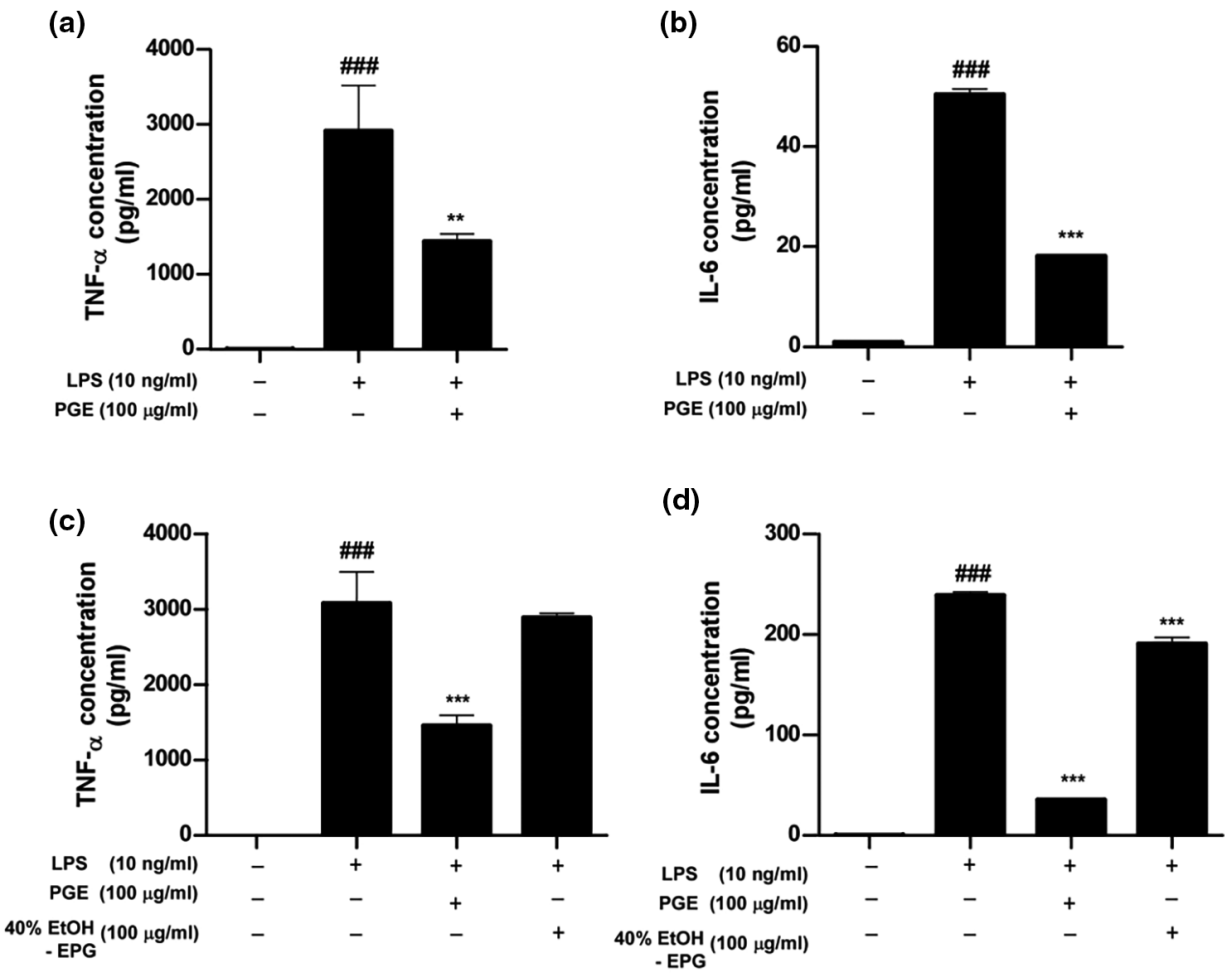
The effect of PGE on the production of TNF‐α and IL‐6 in LPS‐stimulated RAW264.7 murine macrophage cells.

As *P. grandiflorum* has been known to reduce LPS‐induced inflammatory cytokines production, we investigated the effect of PGE on LPS (Lipopolysaccharide)‐induced TNF‐α and IL‐6 levels in RAW264.7 murine macrophage cells (Zhang, Chai, et al., 2022). Our data showed that PGE significantly suppressed the LPS‐induced TNF‐α and IL‐6 levels in RAW264.7 murine macrophage cells at 24 h (Figure 6a,b). A previous study has reported that inhibition of PD on LPS‐induced inflammatory response (Wang et al., 2017) is consistent with our anti‐inflammatory result with high PD content of PGE.

Additionally, we tested the biological activity between PGE optimized in the present study and the extract of PG (EPG) reported previously (Kang et al., 2008; Zhang, Li, et al., 2022), which was extracted with a solvent of 40% ethanol (40% EtOH‐EPG). To compare the biological activity between them, we investigated the effect of PGE and 40% EtOH‐EPG on LPS‐induced TNF‐α and IL‐6 levels in RAW264.7 murine macrophage cells. As shown in Figure 6c,d, PGE more effectively suppresses the LPS‐induced TNF‐α (Figure 6c) and IL‐6 levels (Figure 6b) than 40% EtOH‐EPG. Furthermore, we compared the content of PD contained in PGE and 40% EtOH‐EPG. The content of PD included in 40% EtOH‐EPG was 1.72 mg/g, approximately one‐third of PD content contained in PGE (5.63 mg/g).

Collectively, these results suggest that the biological activity of PGE optimized in the present study has a more effective anti‐inflammatory response compared to 40% EtOH‐EPG, which is consistent with the content of PD in them.

### 3.4 The expectorant activity of PGE

**FIGURE 7:**
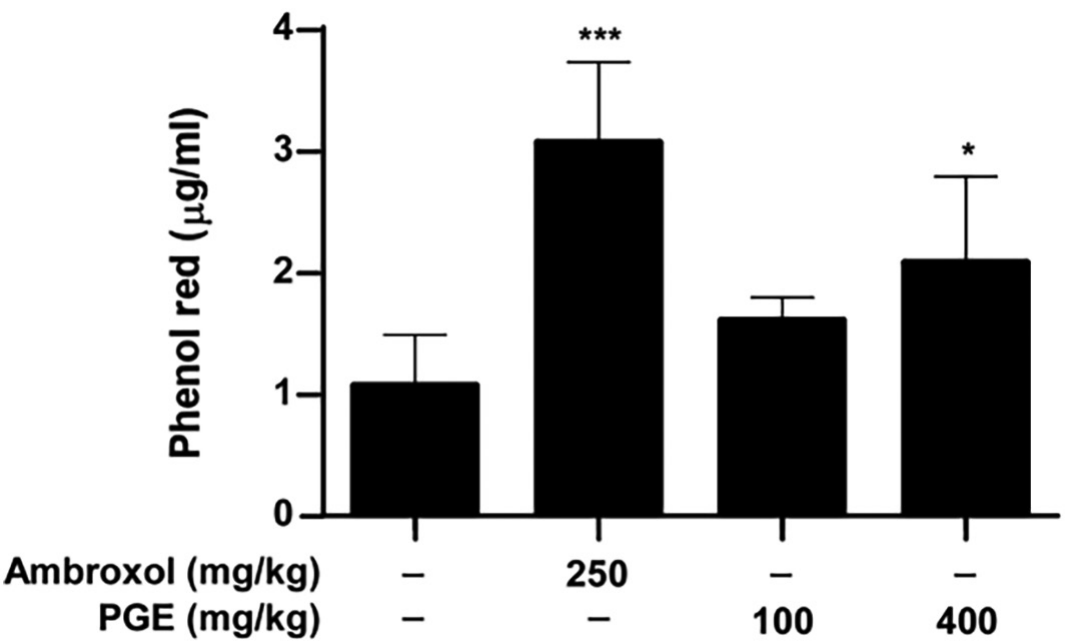
The expectorant effect of PGE.

Expectorant activities were assessed by measuring the amount of phenol red secretion as described in Section 2. As shown in Figure 7, PGE at 400 mg/kg enhanced tracheal phenol red secretion compared with the control group. The Ambroxol was used as a positive control of phenol red secretion (Menezes et al., 2019). This result suggests that PGE might have expectorant effects.

## 4 CONCLUSION

Using the response surface methodology (RSM), the studies on the extraction method to obtain high PD content from PG root have not been well investigated. In the present study, we found the optimum extraction condition as ethanol concentration of 0%, temperature of 50°C, and extraction time of 11 h to obtain PD‐rich *P. grandiflorum* extract (PGE) using RSM. The concentration of PD extracted from the PG root in the optimum condition was close to the PD content predicted by RSM. Additionally, we proved various biological effects of PGE in vitro and vivo, extracted by optimum extraction condition obtained in this study.

These results suggest that this optimal extraction condition will allow PGE to be directly applied to the food industry as food material having therapeutic and preventive potential for human airway diseases.

## AUTHOR CONTRIBUTIONS

**Jihyeon Jung:** Data curation (equal); formal analysis (equal); investigation (equal); methodology (equal); resources (equal); visualization (equal); writing – original draft (equal). **Yeon Jin Cho:** Data curation (equal); formal analysis (equal); investigation (equal); methodology (equal); resources (equal); visualization (equal); writing – original draft (equal). **Minju Jeong:** Data curation (supporting); investigation (supporting); methodology (supporting). **Seung‐Su Lee:** Formal analysis (supporting); methodology (supporting); software (supporting). **Jong Hun Kim:** Data curation (supporting); investigation (supporting); validation (supporting). **Jong‐Eun Kim:** Data curation (supporting); investigation (supporting); validation (supporting). **Nara Kim:** Resources (supporting); software (supporting). **Jiyun Lee:** Methodology (supporting); resources (supporting). **Jung Han Yoon Park:** Conceptualization (supporting); supervision (supporting); validation (supporting). **Ki Won Lee:** Conceptualization (lead); funding acquisition (lead); project administration (lead); supervision (lead); writing – review and editing (supporting). **Sung‐Young Lee:** Conceptualization (lead); data curation (lead); funding acquisition (lead); methodology (lead); project administration (lead); supervision (lead); validation (lead); writing – review and editing (lead).

## CONFLICT OF INTEREST STATEMENT

The authors declare that they have no conflict of interest.

## Data Availability

All data are included in the manuscript.
